# Constitutive Model of Single Root System’s Resistance to Tensile Stress - Taking *Pinus tabulaeformis*, *Betula platyphylla*, *Quercus mongolica* and *Larix gmelinii* as Experimental Objects

**DOI:** 10.1371/journal.pone.0093066

**Published:** 2014-04-15

**Authors:** Lihua Chen, Pinghua Wang, Yuanjun Yang, Jia He

**Affiliations:** Key Laboratory of Soil and Water Conservation & Desertification Combat of Ministry of Education, School of Water and Soil Conservation, Beijing Forest University, Beijing, China; University of Adelaide, Australia

## Abstract

A constitutive model for the stress-strain relationship of single forest root system was developed in order to provide theoretical foundations for the mechanisms of soil-reinforcement by root system and offer a reliable basis for the analysis of root tensile strength character. This study started a general form of linear and non-linear stress-strain relation that was mathematically defined by four boundary conditions observed in typical tensile tests of single roots. The parameters of the model were determined by experiment data and had definite physical meaning. The model was verified by experiment data, which showed that the calculated values were in good agreement with the experimental single root tensile test results. The constitutive model was validated and found to be feasible for modeling single root tensile stress.

## Introduction

Roots are key plant organs that perform vital functions such as absorbing and storing soil moisture, nutrients, and energy, as well as anchoring the plant to the soil. One of the main ecological functions of forest roots is soil stabilization, which can enhance soil conservation, stabilize slopes, prevent gravity erosion, and prevent river and reservoir bank scouring [1]–[4]. Since 1990s, the mechanism of soil-reinforcement by root systems has received widespread attention from research community and led to many interdisciplinary studies [5]–[7]. Since single root of plants is the basic unit of soil-reinforcement, a series of studies has been carried out to study the mechanism of soil-reinforcement by single root system [8].

Currently indoor root tensile tests and *in situ* root-soil composite horizontal and the vertical pullout tests are used to determine the tensile strength, shear strength, and factors contributing to the root system’s resistance to shear of different species [9]–[10]. Some models have been developed to describe the constitutive relationship(the stress-strain relationship) of single root system. Most models use different mathematical functions, such as linear, hyperbolic, polynomial, or power function [11]–[13] to describe the relationship between stress and strain. Those functional constitutive relationships are empirical in nature, since mathematical functions are selected based on the best curve fitting to experimental data. The experiment data determine the constants (or parameters) of the mathematical functions, which do not have clear physical meaning. The applicability and accuracy of those models are greatly limited [13]–[14].

In this study, boundary conditions that a constitutive model for tensile stress of a single root system should conform to are investigated by analyzing typical stress-strain curve of single root systems. A new constitutive model for the tensile stress resistance of single root systems is proposed and mathematically solved using these boundary conditions. Furthermore, the term of critical point (the definition will be presented in Section 2.3) is introduced and derived from the model. Finally, the model is verified by comparing the model calculated values to experimental data, which show good agreement between two. This study may provide theoretical foundations for the mechanisms of soil-reinforcement by root system and give a reliable basis for the analyses of the root tensile strength.

## Materials and Methods

### 2.1 Ethics Statement

The sampling site is managed by Mulan Weichang, a state-owned forest Authority of He-bei province, China. Our study complies with the current laws of China and international rules. All necessary permits have been obtained before the actual sampling. The field study does not involve any endangered or protected species. Data will be made available upon request.

### 2.2 Sample Collection and Testing

The field samples were collected in He-bei province, which surrounds the Capital City of Beijing, at 36° 01′ to 42° 37′ N latitude and 113° 31′ to 119° 53′ E longitude. He-bei province covers a total area of 187,700 square kilometers and has a continental monsoon climate, with cold and dry winters and hot and humid summers. The annual precipitation is 400 to 800 mm, occurring mostly in summer. Roots of four common species of tall trees were chosen to test the proposed model. The trees were *Pinus tabulaeformis*, *Betula platyphylla*, *Quercus mongolica* and *Larix gmelinii*. Tensile tests were carried out for various root diameters and root lengths. To preserve the root and ensure a true reflection of the root mechanical properties, all root samples collected from the field were placed in sealed bags and stored in a refrigerator at 4°C before the test was taking place. All root samples were tested within one month and no pretreatment before testing. 22888 root samples in four gauges (50 mm, 100 mm, 150 mm, and 200 mm) were tested in total; among them, 6462 samples were in gauge 50 mm.

**Figure 1:**
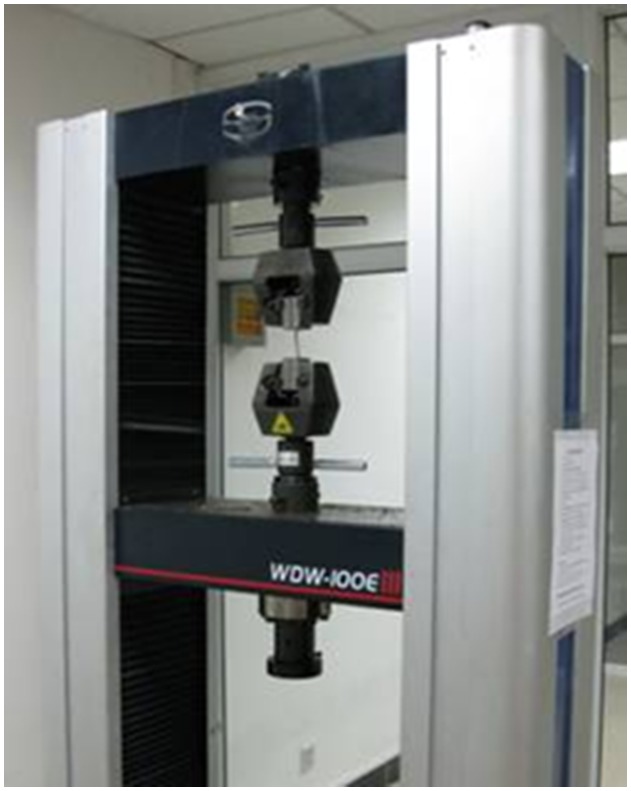
Photo of the universal tester used for tensile test.

Tensile test was performed on WDW-100E electro-universal tester (Time Shijin, China; see Figure 1) equipped with microcomputer control. The test force range was 400–100 kN, and the speed range was 0.001–500 mm/min. The machine has functions of full automatic shift and stepless speed regulation. Its accuracy in measuring test load and displacement is ±0.5%.

This study uses and reports experiment data of single roots with different diameters and fixed gauge (50 mm) only. Root samples with intact bark and relatively uniformed diameter were selected. Each root was divided into four pieces; each piece was roughly 60 mm in length. The diameter at three dividing points was measured and the average diameter was recorded as the root diameter. The universal tester was adjusted to a unified standard distance of 50 mm. Root sample was inserted (50 mm at each end was fixed) onto the instrument and pulled (10 mm/min) until the root is completely pulled apart. Necking was observed during tests. Necking, in engineering and materials science, is a phenomenon that the tensile deformation (strain) has not reached to its extreme when the stress reaches the maximum. The sample statistics of four tree species are summarized in Table 1.

**Table 1:** Summary of sample’s physical features and statistics.

Species	Diameter scope (mm)	Average diameter (mm)	Total root samples	Effective samples	Effective percentage %	Total samples of gauge 50 mm	Effective samples of gauge 50 mm	Effective percentage of gauge 50 mm %
*Pinus*	0.5–1.5	1.11	1539	985	64.00	471	362	76.86
*tabulaeformis*	1.5–2.5	2	2091	1203	57.53	647	341	52.70
	2.5–3.5	3.06	2101	1453	69.16	664	413	62.20
*Larix gmelinii*	3.5–4.5	3.9	2280	1400	61.40	664	278	41.87
	1.5–2.5	2.15	1659	1217	73.36	407	321	78.87
	2.5–3.5	2.99	2291	1782	77.78	741	498	67.21
*Betula*	3.5–4.5	3.98	2164	1225	56.61	702	266	37.89
*platyphylla*	0.5–1.5	1.06	1611	1026	63.69	490	295	60.20
	1.5–2.5	2.41	2500	1178	47.12	526	344	65.40
*Quercus*	2.5–3.5	3.03	1442	781	54.16	463	278	60.04
*mongolica*	1.5–2.5	2.1	1516	872	57.52	687	299	43.52
	3.5–4.5	4.39	1694	855	50.47	594	246	41.41
Total (Average)			22888	13977	61.07	6462	3941	57.35

The test force (stress) and displacement (strain) data were collected by measuring the force and distance required to pull apart a single root. The data were then plotted as stress-strain curves.

### 2.3 Boundary Condition

Before the discussion of the boundary condition, the dimensionless coordinates are defined for convenience:
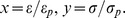
Where 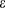 is Strain; 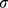 is Stress; 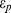 is Peak strain and 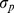 is Peak stress.

The following terms are also used in this study:
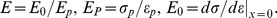
Where *E_0_* is Linear elastic modulus; *E_P_* is Peak secant modulus and *E* is Dimensionless elastic modulus. Since *E_0_* is larger than *E_p_*, *E* is larger than 1.

**Figure 2:**
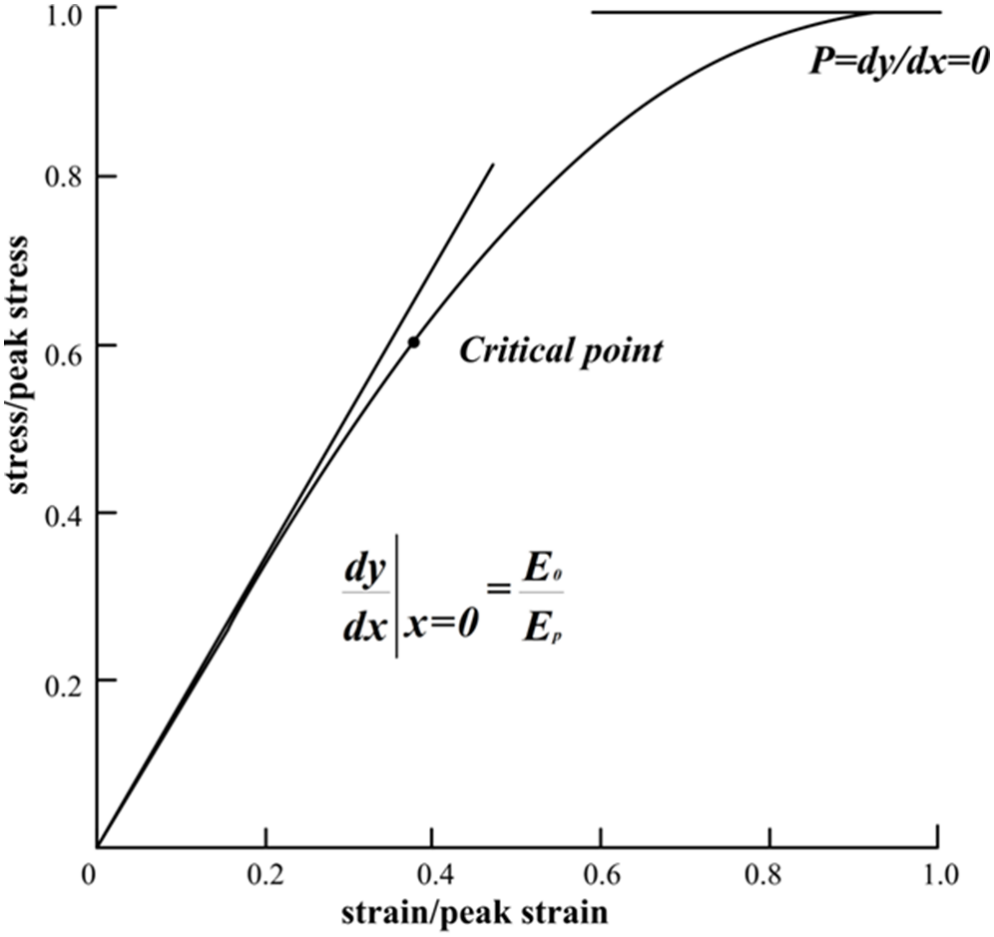
Typical stress-strain curve. (1) *y* = 0, when *x* = 0. The stress-strain curve passes the origin. (2) 
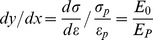
, when *x* = 0. The slope of the curve at the origin is equal to the dimensionless linear elastic modulus of the root. (3) *y* = 1, when *x* = 1. The peak stress happens *x* = 1. (4) 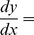
0, when *x* = 1. The slope of the curve at the peak stress is zero.

A typical experiment stress-strain curve of single root is presented in Figure 2. Figure 2 uses dimensionless strain and stress for x and y axis, respectively. It shows that a stress-strain curve starts from zero stress and strain. As the load (stress) increases, the root deformation (strain) increases linearly in the initial stage. As the load continuously increases, the root deformation remain increasing, but the slope of the curve decreases monotonically. The curve is concave down and gradually plateaus to the maximum stress at which the root breaks. A critical point is marked on the curve in Figure 2. Before the critical point, the root linear deformation is dominated and after the critical point, the non-linear deformation becomes significant.

Above observations can be mathematically expressed as:

1) *y* = 0, when *x* = 0. The stress-strain curve passes the origin.
2) 
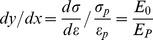
, when *x* = 0. The slope of the curve at the origin is equal to the dimensionless linear elastic modulus of the root.
3) *y* = 1, when *x* = 1. The peak stress happens *x* = 1.
4) 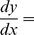
0, when *x* = 1. The slope of the curve at the peak stress is zero.

These four mathematical descriptions are the very boundary conditions that the proposed constitutive model should conform to.

### 2.4 Constitutive Model

As Figure 2 indicates, the root deformation of a single root is composed of both linear and non-linear deformations. Equation (1) is a general form of linear and non-linear deformation of single root.
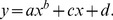
(1)


The four parameters of Equation (1) can be determined by applying the boundary conditions of (1) to (4) described in 2.3. Equation (1) is rewritten as:
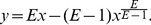
(2)
Equation (2) is our proposed constitutive model for the stress-strain relationship of single root. The first term on the right hand side in Equation (2) describes the linear portion of root deformation, while the second term describes the non-linear portion of root deformation.

The second derivative of Equation (2) is:
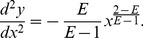
(3)Since *E* is larger than 1, Equation (3) is less than zero, which indicates the curve is concave down, as observed in Figure 2.

As discussed in Section 2.3, the root deformation of single root system is mainly linear in the early stage and non-linear in the late stage, the critical point is the point that separates two stages. The proposed constitutive model of Equation (2) represents both linear and non-linear deformation. Strictly speaking, there is no absolute linear or non-linear deformation. In the early stage, the non-linear deformation, second term of the right hand side of Equation (2), is very small when compared to the linear deformation of the first term. As the curve passes the critical point, the non-linear deformation becomes more and more significant.

### 2.5 Results and Discussion

Data of tensile tests are used to determine physical parameters of single roots, *E_0_* and *E_p_*, used in the proposed constitutive model of Equation (2) for four different root species. The linear elastic modulus (*E_0_*) is the ratio of the material tensile stress to the corresponding tensile strain in the linear range and used to characterize the material resistance to linear deformation capacity. For instance, the linear elastic modulus of *Pinus tabulaeformis* root system is 50% to 70% of the tensile strength limit [15]. It is a safe assumption that the root deformation is mainly linear at 40% of the tensile strength limit for *Pinus tabulaeformis* root system. Therefore, the secant modulus (the ratio of the stress and strain) at 40% of the tensile stress limit was taken as the elastic modulus (*E_0_ = σ_0.4_/ε_0.4_*) for *Pinus tabulaeformis*. The same method is also applied to determine *E_0_* for other three root species. The peak scant modulus, 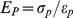, is estimated by the ratio of the measured peak stress and peak strain data.

Traditionally, the root deformation has two stages, linear and non-linear or elastic and plastic deformation. The point that separates the two deformations is called the yield limit. It was commonly accepted that before the yield limit, the root deformation is linear or elastic and after the yield limit, the deformation is non-linear or plastic. However, data of our tensile tests show there is no clear point that separates linear or non-linear deformation. Instead, the dominated linear deformation is observed in early stage of tests, when the stress and strain are small. The weight of non-linear deformation gradually increases. At certain point, the non-linear deformation become significant and cannot be neglected. In this study, the term of the critical point is used to describe that point. Before the critical point, the non-linear deformation is very small when compared to the linear deformation (say less 5% of linear deformation). As the curve passes the critical point, the non-linear deformation becomes more and more significant. The critical point, 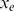, is determined using Equation (2) and corresponding to the 40% of the tensile stress limit.

**Figure 3:**
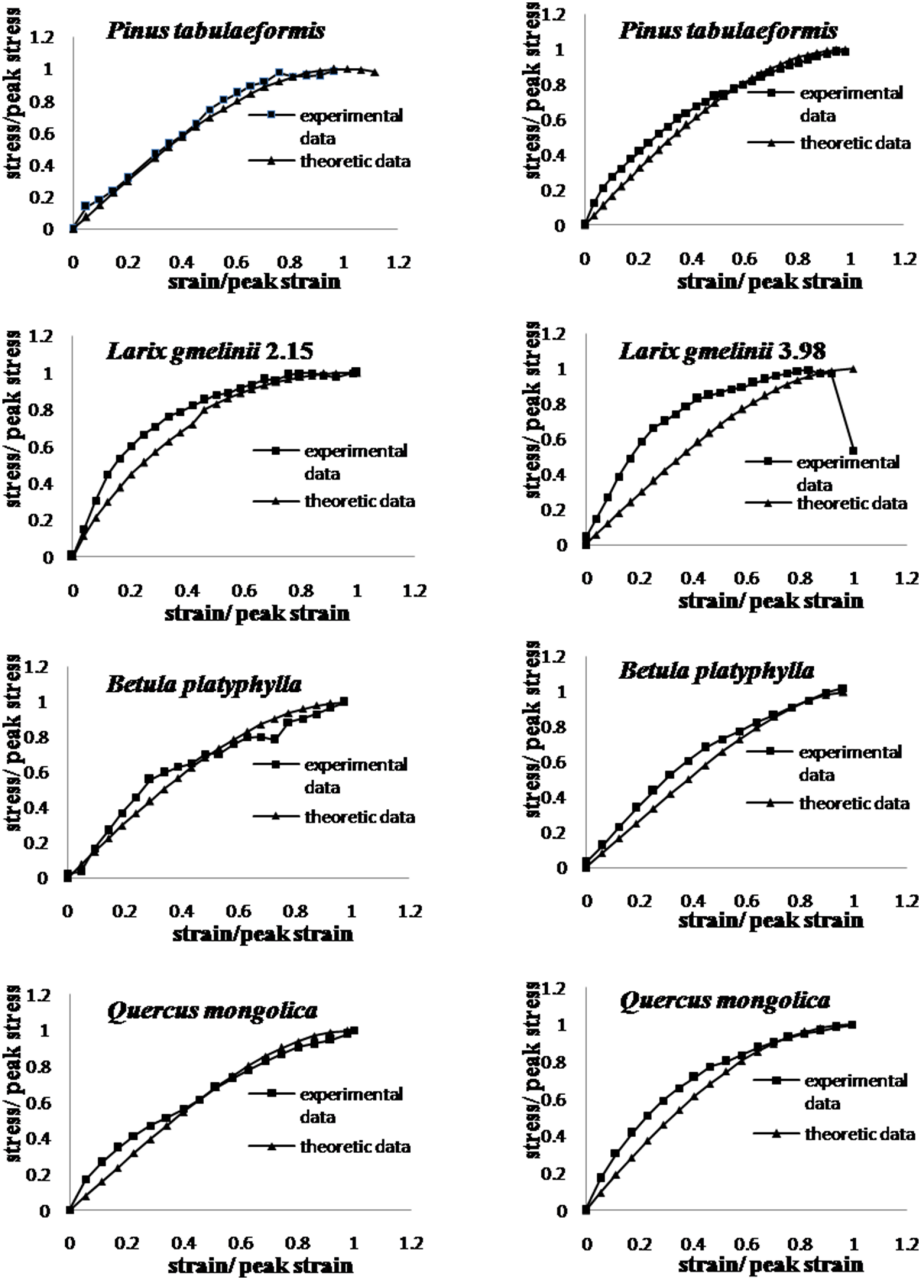
Comparison between model calculated values and experiment data of stress of selected testing. 1.11, 3.06, 2.15, 3.98, 1.06, 3.03 and 2.1 are root diameters. It can be seen that the theoretical stress-strain curves basically fit the experimental curves well.

Once above parameters are determined using experiment data, the proposed model is used to calculate the stress-strain relation. The error between the model calculated values and experiment data is estimated using the residual mean and variance and summarized in Table 2. The most average residual values are less than 0.1 and the most residual variance values are less than 0.01 with only a few exceptions. Figure 3 presents eight figures of model calculated and experiment stress strain data. Seven of eight figures have a good match between model calculated values and experiment data, except the figure for *Latrix gmelinii* (diameter 3.98 mm). Table 2 also shows that an increase in root diameter corresponds to a decrease in elastic modulus (*E_0_*), while the same trend between the root diameter and the peak secant modulus (*E_p_*) is not obvious. As results, the changing trend of *E* versus the root diameter is not clear. Experiment data with different diameters and species may be needed to further verify these observations.

**Table 2:** Model parameters and error results.

Species	Diameter (mm)	Elastic Modulus *E_0_*(MPa)	Peak Secant Modulus E_P_(MPa)	E	CriticalPoint 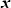 _e_	Average Residuals	Residual Variance
*Pinus tabulaeformis*	1.11	150.00	100.50	1.49241	0.39733	0.02445	0.00095
	2.00	107.12	61.46	1.74292	0.40057	0.06534	0.001366
	3.06	84.06	52.09	1.61375	0.41726	0.03231	0.002625
	3.90	81.06	56.90	1.4246	0.55640	0.081422	0.017675
*Larix gmelinii*	2.15	159.85	80.06	1.99673	0.40986	0.11016	0.006868
	2.99	75.40	39.09	1.92888	0.40330	0.099363	0.013765
	3.98	74.94	51.34	1.45980	0.62916	0.138209	0.01151
*Betula platyphylla*	1.06	183.26	116.93	1.56715	0.40629	0.004367	0.003816
	2.41	180.67	93.98	1.92247	0.40248	0.083052	0.012329
	3.03	180.56	135.70	1.33044	0.59350	0.051374	0.001545
*Quercus mongolica*	2.10	213.27	151.08	1.41163	0.39885	0.016976	0.002841
	4.39	209.96	122.61	1.71242	0.43298	0.057633	0.003057

In summary, a constitutive model for the stress-strain relationship of single roots is proposed in this study. The model starts with a general form of linear and non-linear deformation and is defined with four boundary conditions based on typical stress-strain curves observed from the tensile tests of single roots. The definition of the model requires two physical characteristics of single root: the linear elastic modulus *E_0_* and the peak secant modulus *E_p_*. These two parameters can be obtained from the laboratory tensile strength tests. The major difference between the proposed model and most of previous stress-strain models [13]; [16]–[17] is that the proposed model is based on mathematical expression of physical behavior of single root deformation and boundary conditions of a typical tensile test, instead of on experiment data only. The proposed model has a great potential to help the study of the mechanism of soil-reinforcement by root system.

## Limitations

The mechanism of soil-reinforcement by root systems is a complex issue and factors that may affect it include 1) environmental factors: such as species, direction and position of slope, soil properties, and root moisture content; 2) experimental factors: such as loading speed, loading time, length and diameter of root, storage time of roots and root with or without bark; 3) micro-structural and chemical-composition factors: such as lignin and cellulose content, area ratio of wood fiber and phloem, and length-width ratio of wood fiber and tracheid. Our model developed in this study is based on laboratory root tensile tests. Although the proposed model has clear physical meanings and well defined boundary conditions, it is limited by test data and conditions. Following is discussion about some limitations.

### 3.1 Root Diameters and Tree Species

As discussed above, our tensile tests are carried out in a range of root diameters (1.1 to 4.39 mm) of four tree species (four kinds of tall trees commonly found in Northern China) in one gauge (50 mm) used. The results presented in this paper are limited by those root diameters and tree species. The proposed model needs to be further verified with test data before it is applied to different tree diameters and species and by different methods.

### 3.2 Loading Speed

The loading speed used in our experiments was a constant speed at 10 mm/min. Generally speaking, roots deform elastically before plastically. The root elastic deformation may not be fully developed if the loading speed is too high. Therefore, different loading speeds could affect the stress-strain relationship or the shape of curve. At extreme high loading speed, roots may suffer brittle fracture or necking in early stage of the test.

### 3.3 Storage Time and Moisture Loss

In our experiments, the maximum storage time was 30 days in order to avoid the moisture loss. Our data show that the moisture loss is insignificant within 30 days. The maximum moisture loss was 3% in weight of roots for all roots and 1.4% for roots with diameters larger than 3 mm at the end of 30 days. The moisture loss could be significant after 7 weeks for *Betula platyphylla*, 11 weeks for *Quercus mongolica*, and 8 weeks for *Larix gmelinii* and *Pinus tabulaeformis*.

### 3.4 With or Without Bark

Another reason for the maximum storage time of 30 days is to prevent the deterioration of roots. It is assumed that the deterioration of roots will happen from the bark. Our test samples were with bark and no deterioration was observed in our test samples at the time of testing. The model developed in this paper is applicable for roots with bark. However, root samples with and without bark could make some differences in the stress-strain relationship, which subjects to further study.

### 3.5 Boundary Conditions

**Figure 4:**
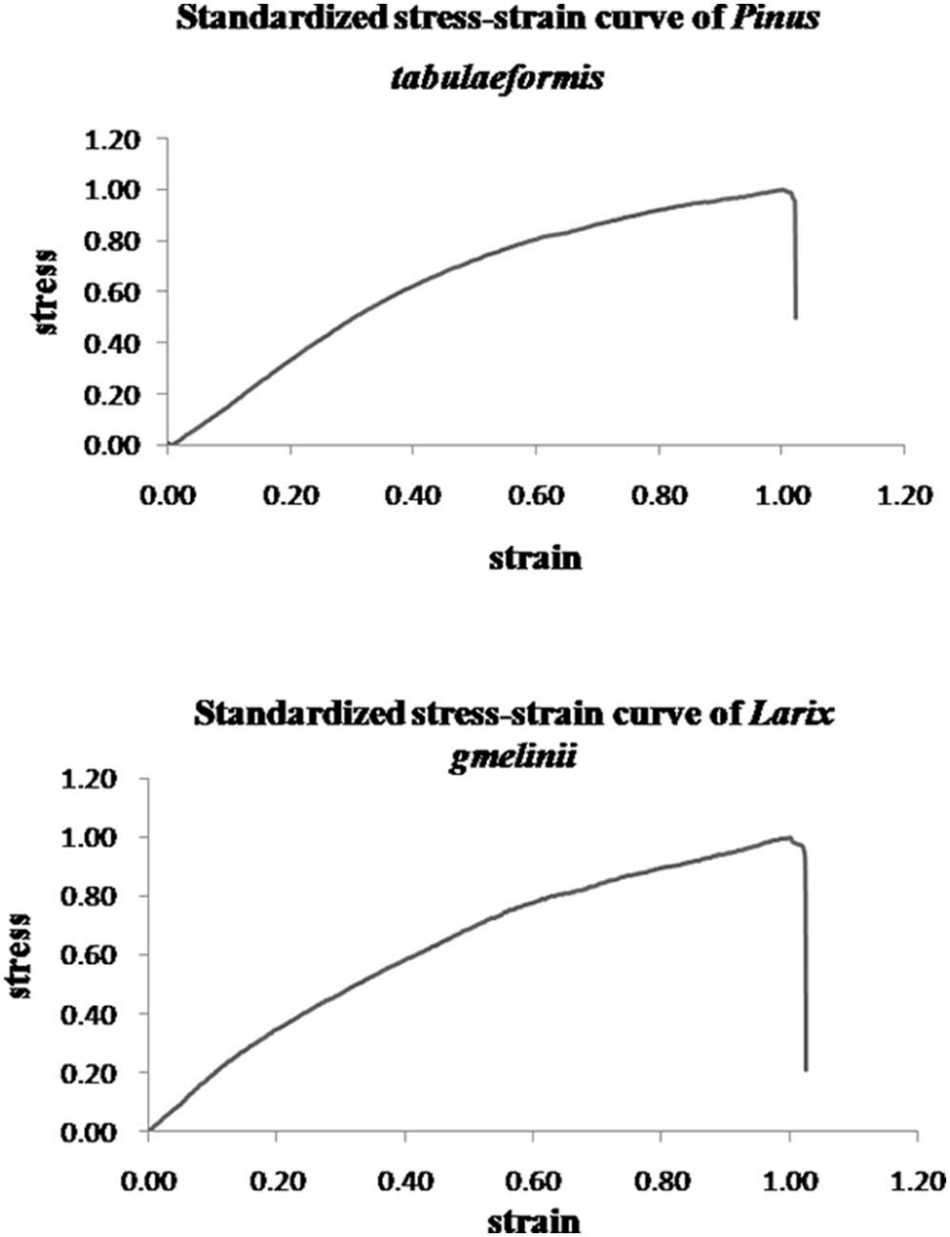
Examples of roots that broke after the stress reached the peak value.

The four boundary conditions discussed in Section 2.3 describe a typical stress-strain relation of single roots, such as observed in our tests. The third boundary condition indicates the peak stress and strain (*y* = 1, when *x* = 1) happen at the same time. The root breaks at the peak stress. This is true for almost all of test samples with a few exceptions. Some of test samples, although the number is small, do show the root deformation continued when the stress reached the peak value or necking. Figure 4 presents two stress-strain curves of such exceptions. It shows the peak stress does not correspond to the peak strain. The value of *x* = 1 does not represent the peak strain, instead it only represents the strain corresponding to the peak stress. The validity of the proposed model for this type of stress-strain relation is to be verified.

Additionally, the elastic modulus, *E_0_*, is determined by the secant modulus at 40% of the tensile stress limit for all four tree species in this study. The selection of the secant modulus is based on previous study in which the elastic modulus of *Pinus tabulaeformis* root system is 50% to 70% of the tensile strength limit. This study applies the same method to determine the elastic modulus of other three tree species. Whether the selection of 40% can be used for determination of the elastic modulus for different tree species used is out of the scope of this study and deserves further study.

Even though with some limitations, the proposed model is promising, since it’s deduced from mathematical procedures and verified by a large amount of test data. The application of the model is simple. It is one of first constitutive models of single root resistance to tensile stress with both physical and mathematical meaning. Systematic and detailed studies on above issues are subjects of further research.

## Conclusions

Although the ultimate aim of our research is to study the role of tree roots in the slope stabilization, the specific aim of this paper is to build a constitutive model of single root system with a clear physical meaning through the rigorous mathematical derivation. The development of the constitutive model and the study of single root physical response to tensile stress are fundamentally significant to the understanding of soil-reinforcement by root systems. Since vegetation is one of major factors that affect slope stability, a common question is how exactly vegetation (especially the mechanical property of roots) affects the soil stabilization. The proposed model of single root resistance to tensile stress and the study reported in this paper provide a theoretical basis for the study of the slope stabilization by vegetation.

The mechanism of soil-reinforcement by root system is investigated by studying single roots of four common tall tree species from Northern China. The data of laboratory tensile tests is used to construct the root tensile constitutive model. The following is a summary of this study:

5) A constitutive model of single root system resistance to tensile stress is proposed.
6)The proposed model is mathematically defined by four boundary conditions observed in a typical tensile test of single root.
7) The term of the critical point is introduced. Before the critical point, the linear deformation is dominated and after the critical point, the non-linear deformation becomes significant.
8) The physical parameters of single root systems, linear elastic modulus (*E_0_*) and peak secant modulus (*E_p_*), can be determined by laboratory tensile tests.
9) Large amount of experiment data from the tensile tests of four common tall trees’ root systems are used for the model verification. The residual mean and variance between the model calculated values and experiment data indicates the proposed model can be used for the study of stress-strain relationships of single roots with good accuracy.
10) The proposed constitutive model of single roots provides theoretical basis for the research of the mechanisms of soil-reinforcement by root system and makes the quantitative analysis of the root tensile strength feasible.
